# Choriocapillaris Vascular Density Changes: Healthy vs. Advanced Exudative Age-Related Macular Degeneration Previously Treated with Multiple Anti-VEGF Intravitreal Injections

**DOI:** 10.3390/diagnostics11111958

**Published:** 2021-10-22

**Authors:** Maria Cristina Savastano, Clara Rizzo, Gloria Gambini, Alfonso Savastano, Benedetto Falsini, Daniela Bacherini, Carmela Grazia Caputo, Raphael Kilian, Francesco Faraldi, Umberto De Vico, Stanislao Rizzo

**Affiliations:** 1 Unit of Ophthalmology, Fondazione Policlinico A. Gemelli, IRCCS, 00191 Rome, Italy; gambini.gloria@gmail.com (G.G.); asavastano21@gmail.com (A.S.); bfalsini@gmail.com (B.F.); carmelagrazia.caputo@gmail.com (C.G.C.); umbertodevico@gmail.com (U.D.V.); stanislao.rizzo@gmail.com (S.R.); 2 Unit of Ophthalmology, Università Cattolica Sacro Cuore, 00168 Rome, Italy; 3 Ophthalmology, Department of Surgical, Medical and Molecular Pathology and Critical Care Medicine, University of Pisa, 56126 Pisa, Italy; 4 Department of Surgery and Translational Medicine, AOU Careggi, University of Florence, 50139 Florence, Italy; daniela.bacherini@gmail.com; 5 Ophthalmology Unit, University of Verona, 37134 Verona, Italy; raphaelkilian8@yahoo.it; 6 Torino, Eye Clinic, ASL Torino 5, 10024 Turin, Italy; faraldi@retinatorino.it; 7 Consiglio Nazionale della Ricerca (CNR), Istituto di Neuroscienze, 56124 Pisa, Italy

**Keywords:** advanced exudative AMD, age-related macular degeneration, innovative biotechnologies, macular neovascularization, OCT angiography, personalized medicine, biomarkers

## Abstract

Purpose: To assess choriocapillaris vascular density (VD) in healthy and advanced exudative age-related macular degeneration (ae-AMD) patients by new full-range optical coherence tomography angiography (OCT-A). Method: In this observational, cross-sectional study, 21 healthy and 21 ae-AMD eyes, already treated with anti-VEGF, were enrolled. Angio-View retina patterns centered on fovea (6.4 × 6.4 mm) were acquired for all participants using Solix full-range OCT (Optovue Inc., Freemont, CA, USA). The main outcome was to compare choriocapillaris VD between healthy and ae-AMD eyes. Automated measurements of whole image choriocapillaris VD (%) and fovea grid-based (%) were collected for the analysis. Angio-View patterns were used to assess the flow area (mm^2^) of macular neovascularization (MNV) by contour flow measure algorithm. Best-corrected visual acuity (BCVA) of both groups was also used for the statistical analysis. Results: The mean age was 60.9 (±8.3) in healthy and 73.33 (±15.05) in ae-AMD eyes. The mean BCVA (ETDRS letters) was 98.47 (±1.50) in healthy and 7.04 (±5.96) in ae-AMD eyes. The Mann–Whitney test comparing choriocapillaries VD for whole and fovea healthy and ae-AMD eyes showed statistical significance (*p* < 0.0001 (t = 4.91; df = 40) and *p* < 0.0001 (t = 6.84; df = 40), respectively). Regarding, the correlation between MNV and VD of choriocapillaries, neither whole nor fovea areas were statistically significant (F = 0.38 (R^2^ = 0.01) and 1.68 (R^2^ = 0.08), respectively). Conclusions: Choriocapillaris VD showed a statistically significant reduction in comparison to healthy eyes in ae-AMD eyes. Choriocapillaris impairment can be seen in the early phase of MNV pathogenesis.

## 1. Introduction

Biomarker identification for age-related macular degeneration is still a challenge for researchers. Recently, the introduction of deep learning in clinical practice allowed to assess the progression of morphological changes through the use of optical coherence tomography (OCT) imaging [1]. While waiting for that deep learning to become easily applicable, clinicians are studying the details derived from innovative imaging modalities. High-definition spectral domain optical coherence tomography angiography (OCT-A) is a relatively new image modality that enables the visualization of the retina and the inner choroid. OCT-A provides structural and vascular information at the same time. Structural OCT has some limitations in distinguishing MNV from drusenoid material or hemorrhages. Fluorescein angiography and indocyanine green angiography have both limitations related to dye injection, i.e., possible adverse events, and the difficulty of visualizing macular neovascularization (MNV) for leakage effect [2]. OCT-A can provide a clean visualization of the MNV silhouette and has several other clinical applications. De Carlo et al. described the qualitative and quantitative characteristics of MNV [3]. OCT-A is also helpful in detecting macular telangiectasia showing the prominent right-angle veins with the distortion of the foveal avascular zone with cavitation [4]. OCT-A, in diabetic retinopathy (DR), shows a larger foveal avascular zone (FAZ) comparing non-proliferative diabetic retinopathy (PDR) with healthy subjects and a wider FAZ in proliferative diabetic retinopathy compared to non-proliferative (NPDR). Moreover, it showed non-perfused areas in deep capillary plexus reflective photoreceptors interruption in diabetic eyes [5].

Since the introduction of OCT-A in clinical practice, we have continued to gain considerable advancements in our knowledge regarding age-related macular degeneration, mostly for the exudative type (AMD). The choriocapillaris is a thin and dense meshwork of large capillaries located in the innermost part of the choroid that serves as the major source of nutrition for the retinal pigment epithelium (RPE) and outer retinal layers. The complex interaction between RPE and choriocapillaris is believed to have a role in the pathogenesis of AMD, and for this reason has been investigated in different studies. It is still not clear if the pathology starts in the RPE or in the choriocapillaris [6].

Jia et al., using a split-spectrum amplitude decorrelation algorithm angiograph (SAADA) algorithm, were able to identify MNV and their precise localization above or below the retinal pigment epithelium (RPE) and the Bruch membrane. Further, close to the MNV, they found an absence of choriocapillaris circulation. They reported that the reduction of choriocapillaris perfusion was not related to geographic atrophy (GA) or to the shadowing effects, but rather to the MNV pathogenesis [7]. Conversely, Invernizzi et al. reported that the choroidal thickening and its vascularity are both secondary effects in eyes with neovascular AMD as reaction of MNV development [8].

Aging is related to a reduction in the choriocapillaris blood flow, which has been speculated to occur due to oxidative stress [9]. The ischemia that follows the oxidative stress is also believed to trigger AMD [10,11].

According to Spaide et al., choriocapillaris blood flow is influenced by age as well [12].

With the advent of OCT-A, choriocapillaris blood flow can be studied in detail to understand its role in pathogenesis of the different AMD forms. In neovascular exudative AMD, the major impairment is due to MNV development. However, choriocapillaris changes can be considered as a landmark, which could be more easily detectable if early disease stages or pathologies with a direct involvement of the choriocapillaris are further investigated. Quantitative vascular density changes in choriocapillaris have been previously detected after anti-VEGF treatment and were defined as a “dark halo”. The fluctuation of choriocapillaris after treatment with anti-VEGF injections may be useful in clinical practice and correspond to a blood sequestering from choriocapillaris by the MNV.

New algorithms have allowed to objectively asses the choriocapillaris vascular density by the post-processing imaging measurements. The aim of our study was to highlight the differences between choriocapillaris VD in healthy ae-AMD eyes by OCT-A.

## 2. Methods

In this observational cross-sectional study, 21 eyes of 21 healthy volunteers and 21 ae-AMD patients, with a mean age of 56.28 (±10.4) and 73.33 (±14.68), respectively, were enrolled.

The study was performed at the Fondazione Policlinico Universitario Agostino Gemelli IRCCS, Rome, Italy between January 2021 and April 2021. The study adhered to the tenets of the Declaration of Helsinki and was approved by the Ethics Committee of the Università Cattolica Sacro Cuore, Rome, Italy (ID number: 3860). Informed consent was obtained before the scanning session. All subjects received a complete ophthalmic examination, which included the measurement of best-corrected visual acuity (BCVA) using the Early Treatment Diabetic Retinopathy Study (ETDRS) chart, slit-lamp biomicroscopy, intraocular pressure (IOP), and dilated funduscopic examination. Inclusion criteria were spherical equivalent refraction within ±3.0 D for both groups. Exclusion criteria were evidence or history of systemic disease with ocular involvement, presence of lens opacities, or signal strength of acquisition scan below 8/10. Exclusion criteria for the healthy control group was the presence of drusen or signs of AMD. All patients in the ae-AMD group already received anti-VEGF therapy, had a mixed type 1 and 2 MNV, and no fluid on the OCT at the moment of the study.

### 2.1. Imaging Protocol and Acquired Data

All images of healthy and pathological eyes were acquired with Solix full-range OCT (Optovue Inc., Freemont, CA, USA), a new ultra-high-speed spectral domain (OCT-A) device (version 2019 V1.0.0.317) that operates at 120,000 A-scans per second with the split spectrum amplitude-decorrelation angiography (SSADA) algorithm. This algorithm, as previously demonstrated, generates a difference between static and non-static tissue (blood flow) that allows the visualization of the vessels by calculating the decorrelation signal amplitude from consecutive B-scans at the same retinal region [13].

Protocol scans used were a high-definition line (b-scan pass through the fovea), a standard multi-volume merge (average four scan volumes) to deliver high-density images with clean clarity, and fundus photography. Before imaging acquisition, each eye was dilated instilling 1% tropicamide eye drops. Study participants underwent scanning protocols consisting of 6.4 × 6.4 mm field of view centered on the fovea. A default internal fixation blue cross light was used to center the scanning area. Two trained operators (MCS and GG) acquired the imaging protocol. The images were acquired between 11 and 12 a.m. to reduce the influence of circadian variation on choriocapillaris.

After completion of the OCT datasets, the embedded software applied Motion Correction Technology, a patented post-processing tool that enables true three-dimensional (3D) correction of distortion in all directions for ultra-precise motion correction. Low-quality scans (i.e., frequent eye blinking or scan with significant motion artifacts) were rejected and repeated acquisition of good-quality scans were achieved for a signal strength ≥ 8/10.

**Figure 1:**
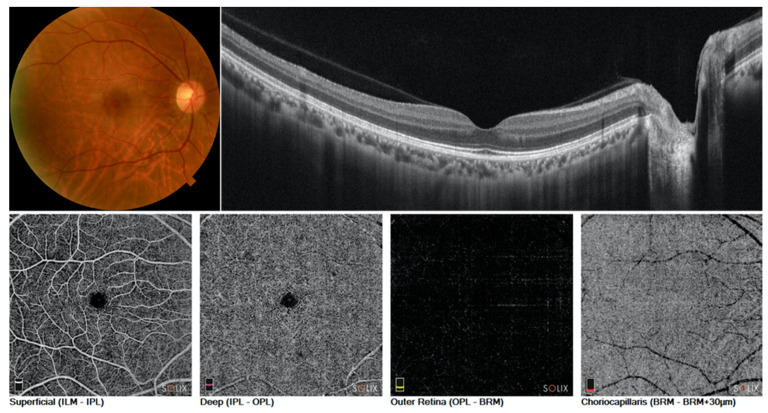
Color fundus photograph (**upper and left**), structural B-scan (**upper and right**), and OCT angiography of superficial, deep, outer, and choriocapillaris (**lower from left to right**) of healthy eye.

**Figure 2:**
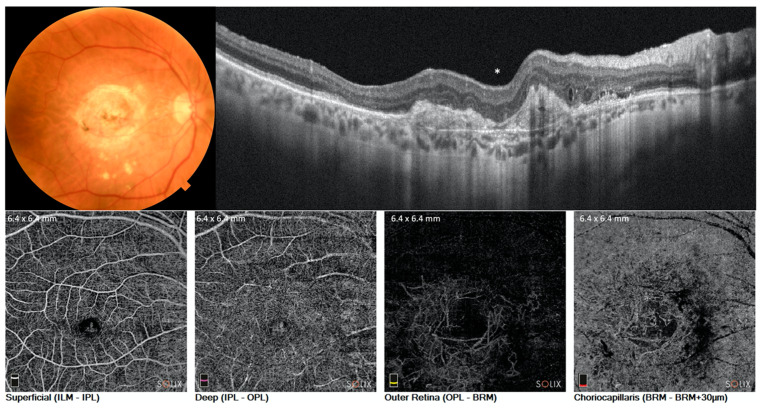
Advanced exudative AMD eye. Color fundus photograph (**upper and left**), shows the scar at the posterior pole. Structural B-scan (**upper and right**) revealed the hyperreflective subfoveal area below the neuroepithelium. OCT angiography of superficial and deep layers was almost preserved, while the outer and choriocapillaris showed macular neovascularization and choriocapillaris defect. * represents the fovea.

Structural OCT and OCT-A images were collected for healthy (Figure 1) and ae-AMD eyes (Figure 2).

The 3D projection artifact removal 2.0 was applied to rapidly remove the projection artifact to simplify image interpretation and to produce more reliable quantification. Before image processing, two retinal specialists (M.C.S. and G.G.), working independently and carefully, visualized all selected images to discern the correctness of the position of the upper and lower boundaries of segmentation corresponding to Bruck’s membrane (BM). If segmentation errors were observed, the user manually corrected a few or all uncorrected segmentations on B-scans and then propagated the correction throughout the entire scan volume. All the choriocapillaris VD evaluations were performed defining setting the set measurement of the slab between 3 µm above the BR and 30 µm below the BM.

**Figure 3:**
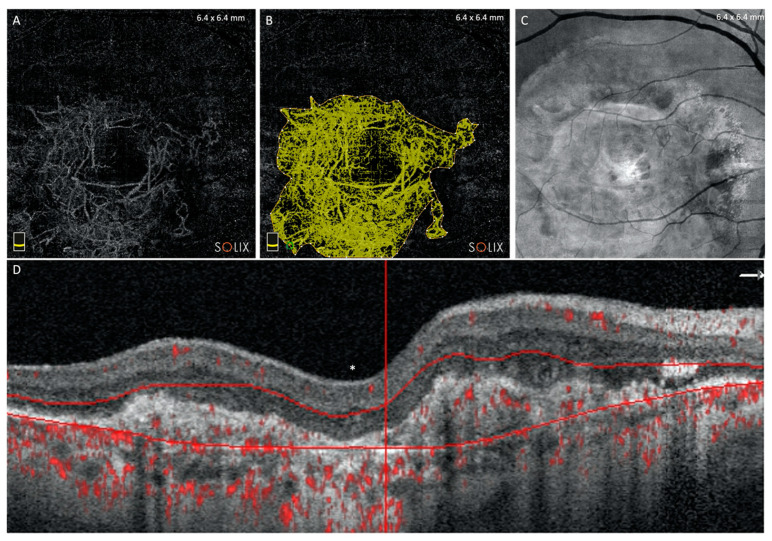
Advanced exudative AMD eye with macular neovascularization (**A**), neovascularization area by semi-automated measurement (yellow area) (**B**), enface and B-scan corresponding in outer retinal slab (**C**,**D**). * represents the fovea.

The AngioView pattern was used to assessed the flow area (mm^2^) of MNV using the contour flow measure algorithm (Figure 3). Two independent operators (M.C.S. and G.G.) analyzed the MNV dimension and the mean of the acquisition was chosen as the final size. The concordance of at least 0.90 was checked by Cohen’s coefficient analysis.

**Figure 4:**
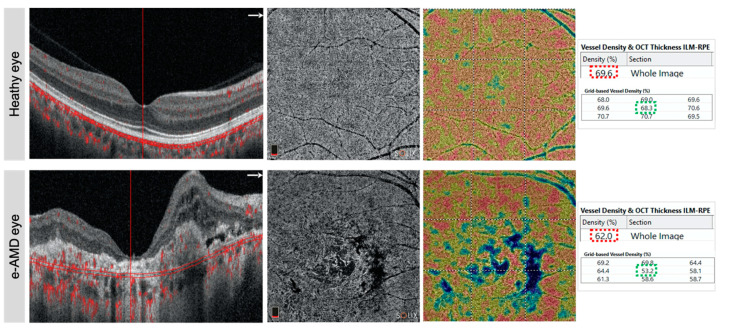
Healthy and advanced exudative AMD eyes details of B-scan (**left**), OCT angiography (**middle**) in correspondence of choriocapillaris, and relative vessel density analysis (**right**). The B-scan shows the flow analysis slab (red line) in choriocapillaris. The automated vessel density in the whole and fovea area were collected for statistical evaluation.

New segmentation algorithms available as a beta version on Solix device, as previously reported, were used to rightly assess choriocapillaris VD layers in heathy and ae-AMD eyes [8,14]. Automated measurements of whole image choriocapillaris VD in percentage (%) and fovea grid-based (%) were collected for the analysis (Figure 4).

### 2.2. Statistical Analysis

The sample size was assessed by a priori sample size estimation using G-Power software package (Version 3.1.9.6). Assuming a minimum difference of 15%, with a residual standard deviation of 10%, a power of 0.8 and an alpha of 0.05 to highlight the differences, the required smallest size was 14 patients for each group. Data were analyzed by GraphPad PRISM Software (Version 8.0; GraphPad, La Jolla, CA, USA). Quantitative variables are presented as mean ± SD. A careful analysis of the data reveled that the log-normal distribution of CVD values obtained in our study population approximated a Gaussian distribution. Therefore, it was justified to apply parametric ANOVA with covariance analysis. The data were analyzed by multivariate analysis of variance (MANOVA), with CC-VD whole and foveal as dependent variables, group (control and patients) as the between-subjects factor, and age as the covariate factor. A *p*-value of less than 0.05 was considered statistically significant.

## 3. Results

A total of 21 healthy eyes and 21 ae-AMD eyes were enrolled in this cross-sectional study. The mean age was respectively 60.9 (±8.3) and 73.33 (±15.05) in healthy and ae-AMD eyes. Demographic and clinical characteristics of the study groups are reported in Table 1.

In Table 2, details of descriptive statistics in the whole and fovea area of choriocapillaris VD (%) are reported. MANOVA comparing CC VD whole and fovea between healthy and ae-AMD showed statistical significance. No significant effect of age as a covariate factor was found. Table 3 reports the results of MANOVA with covariate analysis.

**Figure 5:**
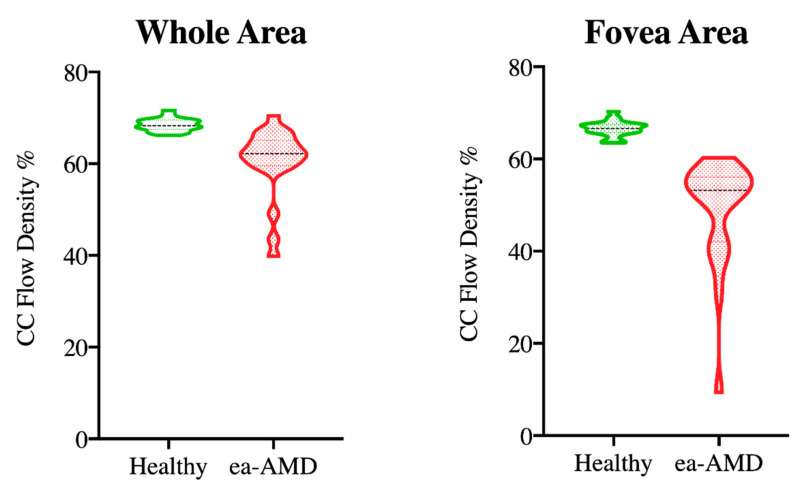
Violin plots show the comparison of choriocapillaris (CC) flow density (%) in healthy and in advanced exudative AMD (ae-AMD) eyes in the whole and fovea area assessed by OCT-Angiography. The plots show the median (black dashed line for heathy and for ae-AMD) and interquartile ranges (red and green lines). The differences were significant (*p* < 0.0001) in both data sets.

Figure 5 shows the plots of comparison between choriocapillaris flow density (%) in the whole and fovea area in healthy and in ae-AMD eyes assessed by OCT-angiography. The differences were significant (*p* < 0.0001) in both datasets.

**Figure 6:**
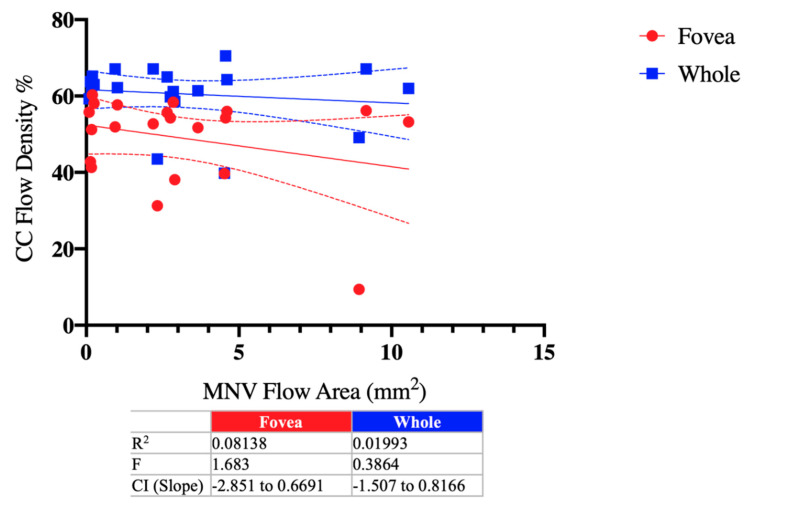
Linear regression between MNV flow area (mm^2^) and choriocapillaris flow density (%) in ae-AMD eyes. No statistical correlation was observed.

The correlations between the MNV area and choriocapillaris vessel density both in the whole and fovea area were not statistically significant and were respectively F = 0.38 (R^2^ = 0.01) and 1.68 (R^2^ = 0.08) (Figure 6).

## 4. Discussion

Advanced imaging analysis allows new methods to study ae-AMD eyes. In our study we evaluated the choriocapillaris vascular density changes in healthy vs. ae-AMD eyes. Our results showed a significant reduction in choriocapillaris vascular density in ae-AMD eyes compared to controls. These findings indicate choriocapillaris vascular density as a possible biomarker in patients with ae-AMD. To the best of our knowledge, this is the first study that evaluates choriocapillaris in advanced AMD eyes.

The choriocapillaris flow is likely to play a dominant role in all forms of macular degeneration, not only exudative forms. Indeed, beneath the area of geographic atrophy (GA), a significant reduction of choriocapillaris perfusion has been observed [15,16]. These hypoperfusion alterations at the level of the choriocapillaris extend beyond the margins of the GA [17,18]. Sacconi et al. reported decreased vessel density at the border of the GA and it has been speculated that the flow impairment on the margin of the GA lesion can predict the direction of the GA expansion [19]. Nassisi et al. performed a flow analysis in patients with GA and found significantly greater flow deficit in the para-atrophy region closer to geographic atrophy compared to the peri-atrophy area. They also found that the severity of blood flow reduction correlates with the enlargement of the GA area and the rapidity of disease progression [20,21]. From what has been investigated, it emerged that the choriocapillaris flow alteration can precede the RPE atrophy, thus making the choriocapillaris alteration a biomarker to predict GA progression [22]. This is in contrast with the theory that emerged from the study by Bhutto et al. speculating that the initial failure in GA seems to start at the level of the RPE whereas the choriocapillaris impairment seems to be a secondary event [23].

A reduction in the choriocapillaris perfusion has also been detected in eyes with intermediate AMD. In these patients, the reduction in choriocapillaris perfusion was more evident in the area surrounding the drusen [24,25]. However, if the overall scan was considered, there was no difference between intermediate AMD patients and healthy controls in terms of choriocapillaris blood flow [26]. In accordance with Luo et al., we found a reduction in choriocapillaris flow in the AMD. Luo et al. mainly evaluated eyes with early and intermediate AMD [27]. In our study, we evaluated advanced non-active AMD patients. Previous findings, in association with our finding of a significant choriocapillaris perfusion impairment between advanced AMD patients and healthy controls, corroborate the hypothesis that the difference in terms of choriocapillaris reflects the stage of the disease, especially because some authors have speculated that the MNV can be considered a last attempt to rescue dying RPE and photoreceptors as a compensatory mechanism to a choriocapillaris deficiency [28].

In agreement with previous studies, Vujosevic et al. found a higher choriocapillaris signal void in patients with intermediate AMD (iAMD) in one eye and neovascular AMD in the other eye [29]. Borrelli et al. observed a correlation between the percent non-perfused choriocapillaris area (PNPCA) and the first negative component (N1) multifocal electroretinogram implicit times in iAMD, thus supporting the hypothesis of an association between choriocapillaris perfusion and photoreceptor function [30]. Considering that patients with neovascular AMD in one eye have a higher risk of developing the same pathology in the fellow eye, the results of the previous studies corroborate the hypothesis that the primum movens of the development of neovascular form of AMD is the choriocapillaris impairment [29]. Corvi et al. confirmed that a high central vascular deficit is a risk factor for AMD progression, and demonstrated that a higher choriocapillaris flow deficiency was independently associated with a higher risk for progression to outer retinal atrophy in AMD (cRORA). Specifically, for each 1% increase in the choriocapillaris flow deficiency, they observed an 11% increase in the risk for progression to complete RPE and cRORA. Moreover, they found that choriocapillaris flow deficiency remained an independent predictor of progression even when other structural OCT biomarkers were considered [31]. Reduction of choriocapillaris flow and vessel density have been observed in patients with reticular pseudodrusen and are associated with greater choriocapillaris loss compared to eyes with other types of drusen. Additionally, the area of choriocapillaris hypoperfusion can extend beyond the area of the pseudodrusen [32,33,34].

Regarding neovascular AMD, a reduction in choriocapillaris blood flow has also been reported [35]. It is interesting that one study noted increased choriocapillaris non-perfusion compared to fellow non-neovascular eyes, implying that choriocapillaris ischemia may play a critical role in the development of these lesions. Furthermore, this study noted greater choriocapillaris non-perfusion in type 3 MNV. Considering the pathogenesis of type 3 MNV, it is particularly interesting to study the choriocapillaris. The speculation is that choriocapillaris non-perfusion may cause biochemical abnormalities of the RPE that lead to the development of type 3 MNV [36]. Because the choriocapillaris is influenced by the VEGF secreted by the RPE, the choriocapillaris blood flow can also be influenced by anti-VEGF therapy.

However, studies on choriocapillaris blood flow and choroidal thickness performed on patients during anti VEGF therapy showed controversial results [37,38,39,40,41]. Hikichi et al. reported a statistically significant decrease in the density of the choriocapillaris during a long-term follow-up of patients with a history of VEGF-treatment [42]. This result can be considered in accordance with the one found by Rispoli et al. In fact, the authors found that during anti-VEGF treatment, there was a reduction in the MNV and in the dark halo surrounding the MNV highlighting that there was also a reduction of the area of choroidal non-perfusion [43]. This dark area seems not to be related to a masking effect but to a real flow reduction, as previously described in a study using indocyanine green angiography (ICG) [44].

It is not clear if the reduction in choriocapillaris blood flow is the predisposing factor for AMD or a consequence of MNV. The region of choroidal neovascularization is often surrounded by localized regions of choriocapillaris non-perfusion. One hypothesis is that ischemia in areas of choriocapillaris flow reduction dictates the location in which neovascular lesions develop; histological reports have shown areas of choriocapillaris atrophy in neovascular AMD eyes [45]. The choriocapillaris showed a greater impairment in the area immediately surrounding the MNV than other areas of the macula [26,46,47]. In fact, in eyes with neovascular, peripheral macula AMD, choriocapillaris perfusion is similar to normal eyes. This is in contrast with the findings of a choriocapillaris impairment in all the macula in patients with GA [48]. This opens two questions; whether the RPE hypoxia drives the release of VEGF and the MNV development or whether the choriocapillaris impairment is secondary to the MNV. Therefore, we probably found a difference between AMD eyes and healthy controls because we selected patients with advanced AMD in contrast with previous studies in which patients with intermediate AMD were selected. In fact, our results are closer to those in patients with GA.

Another hypothesis is that the MNV causes a blood sequestering at the choriocapillaris level. This hypothesis is in accordance with Rispoli et al., who investigated the “dark halo” surrounding the MNV as a marker of its activity. The authors found that the dark halo area shrinks after anti-VEGF treatment and speculated that it could represent an area of secondary ischemia. When the MNV is active, the blood sequestering involved a wider area around the MNV, followed by a choriocapillaris reperfusion and reduction of the dark halo when the MNV is treated with anti-VEGF [36,42].

Our findings of a statistically significant difference in the whole and fovea area of the choriocapillaris perfusion between heathy and ae-AMD patients can be considered a sign of the irreversible choriocapillaris damage. Although possible fluctuations cannot be excluded, it is improbable that they can be considered functional. From our results it emerges that the choriocapillaris damage in the advanced phases of the disease is not related to the dimension of the MNV. Among the results, we also found no correlation between MNV dimension and choriocapillaris impairment, which needs to be interpreted considering that the MNV included in this study were advanced MNV without signs of exudation. We speculate that active MNV can produce a choriocapillaris impairment related to the dimension of the MNV. This study aimed to demonstrate that a non-active neovascular membrane still maintains blood sequestering at the choroidal level. The type and amount of choroidal sequestration can act as a biomarker for other exudative forms.

Our study has some limitations. First of all, choriocapillaris defects can be observable below the intraretinal fluid or near the neuroepithelium detachment. However, none of our cases had exudative signs on the MNV. Another possible artifact could be generated by the presence of pigment deposition into the MNV. Nevertheless, in our cases, we observed the presence of choriocapillaris defects mainly in absence of pigment rearrangement.

In clinical practice the examination and study of choriocapillaris VD could be helpful in the early phases of the choriocapillaris changes. According to the theory that MNV derive from the RPE–choriocapillaris complex protruding into the retina, it is possible that choriocapillaris impairment can be seen as an early phase of the MNV pathogenesis [2,6]. Further studies are needed to evaluate if active MNV causes a major blood sequestering in the choriocapillaris when compared to advanced MNV. Clarifying the role of choriocapillaris in AMD allows to identify choriocapillaris flow as an early biomarker of progression even in other pathologies.

Future applications would be useful to assess choriocapillaris VD in different MNV such as Type 1, Type 2, Combined Type1-Type 2, retinal angiomatous proliferation (RAP), and polypoidal choroidal vasculopathy (PCV).

## Figures and Tables

**Table 1:** Demographic and ophthalmic characteristic of healthy and ae-AMD patients.

Mean Data (±SD)	Healthy	ae-AMD
Age	60.9 (± 8.3)	73.33 (±15.05)
Gender	F/M = 14/7	F/M = 12/9
BCVA (ETDRS letters)	98.47 (±1.50)	7.04 (±5.96)
Spherical equivalent	−0.09 (±1.02)	−0.1 (±1)
MNV flow area (mm^2^)	0	3.08 (±3.13)
CC flow density (whole)	68.48 (±1.4)	60.59 (±7.8)
CC flow density (fovea)	66.6 (±2.2)	49.05 (±12.2)

AMD: advanced exudative age-related macular degeneration; CC: choriocapillaris; SD: standard deviation.

**Table 2:** Descriptive statistics for the whole and fovea area of choriocapillaris vascular density (%).

	Whole		Fovea	
	Healthy	ae-AMD	Healthy	ae-AMD
Number of values	21	21	21	21
Minimum	66.2	39.8	63.5	9.4
Maximum	71.6	70.5	70.3	60.3
Mean	68.48	60.6	66.6	49.05
Std. Error of Mean	0.3030	1.667	0.3666	2.609
Lower 95% CI	67.85	57.12	65.83	43.61
Upper 95% CI	69.11	64.07	67.36	54.49
Mann–Whitney test	*p* < 0.0001 (t = 4.91; df =4 0)		*p* < 0.0001	
			(t = 6.84; df = 40)	

**Table 3:** MANOVA comparing CC VD whole and fovea between healthy and ae-AMD showed statistical significance. No significant effect of age as a covariate factor was found.

Multivariate Tests ^b^							
Effect		Value	F	Hypothesis df	Error df	Sig.	Partial Eta Squared
Intercept	Pillai’s Trace	0.838	98,515 ^a^	2000	38,000	0.000	0.838
	Wilks’ Lambda	0.162	98,515 ^a^	2000	38,000	0.000	0.838
	Hotelling’s Trace	5185	98,515 ^a^	2000	38,000	0.000	0.838
	Roy’s Largest Root	5185	98,515 ^a^	2000	38,000	0.000	0.838
AGE (covariate)	Pillai’s Trace	0.040	0.784 ^a^	2000	38,000	0.464	0.040
	Wilks’ Lambda	0.960	0.784 ^a^	2000	38,000	0.464	0.040
	Hotelling’s Trace	0.041	0.784 ^a^	2000	38,000	0.464	0.040
	Roy’s Largest Root	0.041	0.784 ^a^	2000	38,000	0.464	0.040
Group	Pillai’s Trace	0.438	14,836 ^a^	2000	38,000	0.000	0.438
	Wilks’ Lambda	0.562	14,836 ^a^	2000	38,000	0.000	0.438
	Hotelling’s Trace	0.781	14,836 ^a^	2000	38,000	0.000	0.438
	Roy’s Largest Root	0.781	14,836 ^a^	2000	38,000	0.000	0.438

^a^ Exact statistic, ^b^ Design: Intercept + VAR00002 + VAR00001.

## Data Availability

Data are available at the following link: https://www.dropbox.com/home/CVI?oref=e&preview=CVI+Advanced+AMD+%28WET%29.xlsx+1.xlsx (accessed on 1 January 2021).

## References

[1] Vogl W.-D., Bogunović H., Waldstein S.M., Riedl S., Schmidt-Erfurth U. (2021). Spatio-Temporal Alterations in Retinal and Choroidal Layers in the Progression of Age-Related Macular Degeneration (AMD) in Optical Coherence Tomography. Sci. Rep..

[2] De Carlo T.E., Romano A., Waheed N.K., Duker J.S. (2015). A review of optical coherence tomography angiography (OCTA). Int. J. Retina Vitreous.

[3] de Carlo T.E., Bonini Filho M.A., Chin A.T., Adhi M., Ferrara D., Baumal C.R., Witkin A.J., Reichel E., Duker J.S., Waheed N.K. (2015). Spectral-domain optical coherence tomography angiography of choroidal neovascularization. Ophthalmology.

[4] Rocholz R., Corvi F., Weichsel J., Schmidt S., Staurenghi G., Bille J.F. (2019). OCT Angiography (OCTA) in Retinal Diagnostics. 14 August 2019. High Resolution Imaging in Microscopy and Ophthalmology: New Frontiers in Biomedical Optics [Internet].

[5] Tey K.Y., Teo K., Tan A.C.S., Devarajan K., Tan B., Tan J., Schmetterer L., Ang M. (2019). Optical coherence tomography angiography in diabetic retinopathy: A review of current applications. Eye Vis..

[6] Manian K.V., Galloway C.A., Dalvi S., Emanuel A.A., Mereness J.A., Black W., Winschel L., Soto C., Li Y., Song Y. (2021). 3D IPSC Modeling of the Retinal Pigment Epithelium-Choriocapillaris Complex Identifies Factors Involved in the Pathology of Macular Degeneration. Cell Stem Cell.

[7] Jia Y., Bailey S.T., Wilson D.J., Tan O., Klein M.L., Flaxel C.J., Potsaid B., Liu J.J., Lu C.D., Kraus M.F. (2014). Quantitative optical coherence tomography angiography of choroidal neovascularization in age-related macular degeneration. Ophthalmology.

[8] Invernizzi A., Benatti E., Cozzi M., Erba S., Vaishnavi S., Vupparaboina K.K., Staurenghi G., Chhablani J., Gillies M., Viola F. (2018). Choroidal Structural Changes Correlate With Neovascular Activity in Neovascular Age Related Macular Degeneration. Investig. Ophthalmol. Vis. Sci..

[9] Chirco K.R., Sohn E.H., Stone E.M., Tucker B.A., Mullins R.F. (2017). Structural and Molecular Changes in the Aging Choroid: Implications for Age-Related Macular Degeneration. Eye.

[10] Boltz A., Luksch A., Wimpissinger B., Maar N., Weigert G., Frantal S., Brannath W., Garhofer G., Ergun E., Stur M. (2010). Choroidal Blood Flow and Progression of Age-Related Macular Degeneration in the Fellow Eye in Patients with Unilateral Choroidal Neovascularization. Investig. Ophthalmol. Vis. Sci..

[11] Feigl B. (2009). Age-Related Maculopathy—Linking Aetiology and Pathophysiological Changes to the Ischaemia Hypothesis. Prog. Retin. Eye Res..

[12] Spaide R.F. (2016). Choriocapillaris Flow Features Follow a Power Law Distribution: Implications for Characterization and Mechanisms of Disease Progression. Am. J. Ophthalmol..

[13] Jia Y., Tan O., Tokayer J., Potsaid B., Wang Y., Liu J.J., Kraus M.F., Subhash H., Fujimoto J.G., Hornegger J. (2012). Split-Spectrum Amplitude-Decorrelation Angiography with Optical Coherence Tomography. Opt. Express.

[14] Di Antonio L., Viggiano P., Ferro G., Toto L., D’Aloisio R., Porreca A., Di Nicola M., Mastropasqua R. (2020). Retinal Vascular Metrics Difference by Comparison of Two Image Acquisition Modes Using a Novel OCT Angiography Prototype. PLoS ONE.

[15] Moult E.M., Waheed N.K., Novais E.A., Choi W., Lee B., Ploner S.B., Cole E.D., Louzada R.N., Lu C.D., Rosenfeld P.J. (2016). Swept-source optical coherence tomography angiography reveals choriocapillaris alterations in eyes with nascent geographic atrophy and drusen-associated geographic atrophy. Retina.

[16] Arya M., Sabrosa A.S., Duker J.S., Waheed N.K. (2018). Choriocapillaris Changes in Dry Age-Related Macular Degeneration and Geographic Atrophy: A Review. Eye Vis..

[17] Choi W., Moult E.M., Waheed N.K., Adhi M., Lee B., Lu C.D., de Carlo T.E., Jayaraman V., Rosenfeld P.J., Duker J.S. (2015). Ultrahigh-Speed, Swept-Source Optical Coherence Tomography Angiography in Nonexudative Age-Related Macular Degeneration with Geographic Atrophy. Ophthalmology.

[18] Kvanta A., Casselholm de Salles M., Amrén U., Bartuma H. (2017). Optical coherence tomography angiography of the foveal microvasculature in geographic atrophy. Retina.

[19] Sacconi R., Corbelli E., Carnevali A., Querques L., Bandello F., Querques G. (2018). Optical coherence tomography angiography in geographic atrophy. Retina.

[20] Nassisi M., Shi Y., Fan W., Borrelli E., Uji A., Ip M.S., Sadda S.R. (2019). Choriocapillaris Impairment around the Atrophic Lesions in Patients with Geographic Atrophy: A Swept-Source Optical Coherence Tomography Angiography Study. Br. J. Ophthalmol..

[21] Nassisi M., Baghdasaryan E., Borrelli E., Ip M., Sadda S.R. (2019). Choriocapillaris Flow Impairment Surrounding Geographic Atrophy Correlates with Disease Progression. PLoS ONE.

[22] Lindner M., Böker A., Mauschitz M.M., Göbel A.P., Fimmers R., Brinkmann C.K., Schmitz-Valckenberg S., Schmid M., Holz F.G., Fleckenstein M. (2015). Directional Kinetics of Geographic Atrophy Progression in Age-Related Macular Degeneration with Foveal Sparing. Ophthalmology.

[23] Bhutto I., Lutty G. (2012). Understanding Age-Related Macular Degeneration (AMD): Relationships between the Photoreceptor/Retinal Pigment Epithelium/Bruch’s Membrane/Choriocapillaris Complex. Mol. Aspects Med..

[24] Borrelli E., Uji A., Sarraf D., Sadda S.R. (2017). Alterations in the Choriocapillaris in Intermediate Age-Related Macular Degeneration. Investig. Ophthalmol. Vis. Sci..

[25] Borrelli E., Shi Y., Uji A., Balasubramanian S., Nassisi M., Sarraf D., Sadda S.R. (2018). Topographic Analysis of the Choriocapillaris in Intermediate Age-Related Macular Degeneration. Am. J. Ophthalmol..

[26] Alagorie A.R., Verma A., Nassisi M., Nittala M., Velaga S., Tiosano L., Sadda S.R. (2020). Quantitative assessment of choriocapillaris flow deficits surrounding choroidal neovascular membranes. Retina.

[27] Luo M., Zhao X., Zhao N., Yuan M., Yang J., Dai R., Chen Y. (2020). Comparison of choriocapillary flow density between fellow eyes of polypoidal choroidal vasculopathy and neovascular age-related macular degeneration. BMC Ophthalmol..

[28] Christenbury J.G., Phasukkijwatana N., Gilani F., Freund K.B., Sadda S., Sarraf D. (2018). Progression of macular atrophy in eyes with type 1 neovascularization and age-related macular degeneration receiving long-term intravitreal anti-vascular endothelial growth factor therapy: An Optical Coherence Tomographic Angiography Analysis. Retina.

[29] Vujosevic S., Toma C., Villani E., Muraca A., Torti E., Florimbi G., Pezzotti M., Nucci P., De Cillà S. (2019). Quantitative Choriocapillaris Evaluation in Intermediate Age-related Macular Degeneration by Swept-source Optical Coherence Tomography Angiography. Acta Ophthalmol..

[30] Borrelli E., Mastropasqua R., Senatore A., Palmieri M., Toto L., Sadda S.R., Mastropasqua L. (2018). Impact of Choriocapillaris Flow on Multifocal Electroretinography in Intermediate Age-Related Macular Degeneration Eyes. Investig. Ophthalmol. Vis. Sci..

[31] Corvi F., Tiosano L., Corradetti G., Nittala M.G., Lindenberg S., Alagorie A.R., McLaughlin J.A., Lee T.K., Sadda S.R. (2021). Choriocapillaris flow deficits as a risk factor for progression of age-related macular degeneration. Retina.

[32] Cicinelli M.V., Rabiolo A., Marchese A., de Vitis L., Carnevali A., Querques L., Bandello F., Querques G. (2017). Choroid Morphometric Analysis in Non-Neovascular Age-Related Macular Degeneration by Means of Optical Coherence Tomography Angiography. Br. J. Ophthalmol..

[33] Nesper P.L., Soetikno B.T., Fawzi A.A. (2017). Choriocapillaris Nonperfusion Is Associated With Poor Visual Acuity in Eyes With Reticular Pseudodrusen. Am. J. Ophthalmol..

[34] Chatziralli I., Theodossiadis G., Panagiotidis D., Pousoulidi P., Theodossiadis P. (2018). Choriocapillaris’ Alterations in the Presence of Reticular Pseudodrusen Compared to Drusen: Study Based on OCTA Findings. Int. Ophthalmol..

[35] Borrelli E., Sarraf D., Freund K.B., Sadda S.R. (2018). OCT Angiography and Evaluation of the Choroid and Choroidal Vascular Disorders. Prog. Retin Eye Res..

[36] Borrelli E., Souied E.H., Freund K.B., Querques G., Miere A., Gal-Or O., Sacconi R., Sadda S.R., Sarraf D. (2018). Reduced choriocapillaris flow in eyes with type 3 neovascularization and age-related macular degeneration. Retina.

[37] Yamazaki T., Koizumi H., Yamagishi T., Kinoshita S. (2012). Subfoveal Choroidal Thickness after Ranibizumab Therapy for Neovascular Age-Related Macular Degeneration: 12-Month Results. Ophthalmology.

[38] Ting D.S.W., Yanagi Y., Agrawal R., Teo H.Y., Seen S., Yeo I.Y.S., Mathur R., Chan C.M., Lee S.Y., Wong E.Y.M. (2017). Choroidal Remodeling in Age-Related Macular Degeneration and Polypoidal Choroidal Vasculopathy: A 12-Month Prospective Study. Sci. Rep..

[39] Lipecz A., Miller L., Kovacs I., Czakó C., Csipo T., Baffi J., Csiszar A., Tarantini S., Ungvari Z., Yabluchanskiy A. (2019). Microvascular Contributions to Age-Related Macular Degeneration (AMD): From Mechanisms of Choriocapillaris Aging to Novel Interventions. GeroScience.

[40] Govetto A., Sarraf D., Figueroa M.S., Pierro L., Ippolito M., Risser G., Bandello F., Hubschman J.P. (2017). Choroidal Thickness in Non-Neovascular versus Neovascular Age-Related Macular Degeneration: A Fellow Eye Comparative Study. Br. J. Ophthalmol..

[41] Minnella A.M., Federici M., Falsini B., Barbano L., Gambini G., Lanza A., Caporossi A., Savastano M.C. (2016). Choroidal Thickness Changes After Intravitreal Ranibizumab for Exudative Age-Related Macular Degeneration. BioDrugs.

[42] Hikichi T., Agarie M. (2019). Reduced Vessel Density of the Choriocapillaris during Anti-Vascular Endothelial Growth Factor Therapy for Neovascular Age-Related Macular Degeneration. Investig. Ophthalmol. Vis. Sci..

[43] Rispoli M., Savastano M.C., Lumbroso B. (2018). Quantitative Vascular Density Changes in Choriocapillaris Around CNV After Anti-VEGF Treatment: Dark Halo. Ophthalm. Surg. Lasers Imaging Retin..

[44] Gharbiya M., Pantaleoni F.B., Grandinetti F., Gabrieli C.B. (1999). Indocyanine Green Angiographic Findings in Idiopathic Choroidal Neovascularisation. Eye.

[45] McLeod D.S., Taomoto M., Otsuji T., Green W.R., Sunness J.S., Lutty G.A. (2002). Quantifying Changes in RPE and Choroidal Vasculature in Eyes with Age-Related Macular Degeneration. Investig. Ophthalmol. Vis. Sci..

[46] Treister A.D., Nesper P.L., Fayed A.E., Gill M.K., Mirza R.G., Fawzi A.A. (2018). Prevalence of Subclinical CNV and Choriocapillaris Nonperfusion in Fellow Eyes of Unilateral Exudative AMD on OCT Angiography. Transl. Vis. Sci. Technol..

[47] Moult E.M., Alibhai A.Y., Rebhun C., Lee B., Ploner S., Schottenhamml J., Husvogt L., Baumal C.R., Witkin A.J., Maier A. (2020). Spatial distribution of choriocapillaris impairment in eyes with choroidal neovascularization secondary to age-related macular degeneration: A Quantitative OCT Angiography Study. Retina.

[48] Scharf J., Corradetti G., Corvi F., Sadda S., Sarraf D. (2021). Optical Coherence Tomography Angiography of the Choriocapillaris in Age-Related Macular Degeneration. JCM.

